# Factors associated with immediate postoperative pulmonary complications after Appendectomies under general anesthesia: A retrospective analysis

**DOI:** 10.5339/qmj.2023.20

**Published:** 2023-12-12

**Authors:** Neeraj Kumar, Mohammed AM Ayasa, Chitrambika P Krishnadas, Prem Chandra, Mamdouh M Al-Mustafa, Simi Praveen, Tripti Sinha, Sreethish Sasi

**Affiliations:** ^1^Department of Anesthesiology, Hamad Medical Corporation, Doha, Qatar Email: CDas3@hamad.qa; ^2^Weill Cornell Medical College, Qatar; ^3^Medical Research Centre, Hamad Medical Corporation, Doha, Qatar; ^4^Department of Anesthesiology, Sidra Medicine, Doha, Qatar; ^5^Infectious Diseases Division, Department of Internal Medicine, Hamad Medical Corporation, Doha, Qatar

**Keywords:** Appendicitis, Appendectomy, Post-operative, Complications, General Anesthesia

## Abstract

Background: Postoperative pulmonary complications (PPC) include any complication that affects the respiratory system after anesthesia and surgery and are a significant cause of postoperative mortality and morbidity.

Objectives: To describe the risk factors for immediate postoperative pulmonary complications after appendectomy under general anesthesia and to determine if rapid sequence induction decreases the risk.

Design and Setting: A retrospective analysis of perioperative medical records of patients who underwent appendectomy under general anesthesia over a year, from January 1st, 2014, to December 31st, 2014, at Hamad General Hospital, Doha, Qatar, was done.

Results: Of the 1005 patients who met the inclusion criteria, 27 (3.7%) had PPC. The incidence of PPC had a significant positive association with diabetes mellitus (DM), bronchial asthma (BA), number of intubation attempts, laparoscopic approach, and longer surgeries (>2 h). Hypertension, recent or ongoing upper respiratory tract infections, and smoking were not associated with an increased risk of PPC. Non-rapid sequence intubation (RSI) was not associated with an increased risk of PPC compared with RSI.

Conclusions: The incidence of immediate PPC in ASA 1 and 2 appendectomy patients aged between 15 and 50 is significant. There is an increased risk among asthmatics, diabetics, and those with difficult airways. The RSI technique does not offer protection.

## Introduction

Acute appendicitis is among the most common causes of emergency abdominal surgery.^1^ Worldwide, the total pooled incidence of appendicitis or appendectomy is around 100–150 per 100,000 person-years.^2^ Recent studies suggest a rise in the prevalence of appendicitis in developing countries between the early teenage years and the late 40s.^2^ A higher prevalence is seen in men than in women. The disease outcome is affected by its natural course and the risks and complications of treatment.^2^ Postoperative pulmonary complications (PPC) include almost any complication that affects the respiratory system after anesthesia and surgery. These complications are highly prevalent, significantly impact patient care, and are difficult to predict.^3^ The incidence of PPCs in major surgery ranges from less than 1% to 23% and has a high rate of association with morbidity, mortality, and the duration of hospital stay.^3–5^ The most severe PPCs are atelectasis, pneumonia, respiratory failure, and underlying chronic lung disease exacerbations.^6^ A systematic review by the American College of Physicians demonstrated that 60% of studies used a combination of pneumonia and respiratory failure to define PPCs.^6^ Emergency procedures and abdominal surgeries have a higher risk of regurgitation and aspiration.^7^ There is a lower risk of PPC in patients undergoing emergency appendectomy (laparoscopic or open) with rapid sequence induction (RSI) compared to non-RSI patients.^8^

In 2015, a European joint task force published guidelines for the perioperative clinical outcome (EPCO), defining pneumonia, acute respiratory distress syndrome (ARDS), and pulmonary embolism (PE) as individual adverse events and considering respiratory infection, respiratory failure, pleural effusion, atelectasis, pneumothorax, bronchospasm, and aspiration pneumonitis to be the parameters.^3,9^ The precise outcome measures concerning PPC and associated risk factors in appendectomy patients have yet to be extensively studied. The primary aim of this study was to assess and identify risk factors for PPCs in patients undergoing appendectomies under general anesthesia. The secondary objective is to determine the difference in risk between RSI and non-RSI of general anesthesia in causing PPC. We believe this knowledge will help identify and better manage those at increased risk of PPC after appendectomy.

## Methods

### Patients

Ethical approval for this study (MRC/0895/2015) was provided by the Institutional Review Board (IRB) at the Medical Research Center (MRC) of Hamad Medical Corporation, Doha, Qatar (Chairperson: Dr. Ali Omrani), on September 16^th^, 2015. This is a retrospective review of the anesthetic and perioperative records of patients who underwent appendectomy under general anesthesia over one year, from January 1^st^, 2014, to December 31st, 2014. Patients with preexisting hypoxia, pneumonia, surgery under spinal anesthesia, aged less than 15 years or more than 50, and ASA physical status 3 or 4 were excluded from the study. Standard demographic data were collected regarding age, gender, and nil-per-orals (NPO) duration. Pre-existing comorbidities included bronchial asthma (BA), diabetes mellitus (DM), hypertension (HTN), recent or ongoing upper respiratory tract infections (URTI), and a history of smoking. Intraoperative data was collected from the anesthetic charts. All intra-operative data that was collected is summarized in Table 1. The presence of PPC was assessed by the data collected from PACU. These post-operative parameters are summarized in Table 2.

### Statistical Analysis

Descriptive statistics were used to summarize demographic, clinical, surgical, and post-surgical outcomes, including PPC and other related characteristics of the participants. The normally distributed data and results were reported with a mean and standard deviation (SD). The remaining results were reported with a median and interquartile range (IQR). Categorical data values were expressed as frequencies and percentages. Associations between two or more qualitative variables were evaluated using the Chi-square (X^2^) test or Fisher exact test, as appropriate. This study's secondary outcome measures were looking for PPC differences between RSI and non-RSI under general anesthesia and the most common postoperative complications in the open and laparoscopic appendectomy groups. These were statistically compared and analyzed using the statistical methods stated above. Univariate and multivariate logistic regression analysis was applied to determine and assess the associations of potential predictors and risk factors (such as the presence of DM, intubation attempts, length of surgery, surgical technique, the reversal of neuromuscular blocker (NMB), and RSI vs. non-RSI anesthesia induction) associated with PPC. The results of logistic regression analyses were expressed as odds ratios (OR) with corresponding 95% confidence intervals (CI). All p-values presented were two-tailed; p-values less than 0.05 were considered statistically significant. All statistical analyses were done using the statistical packages SPSS 22.0 (SPSS Inc., Chicago, IL) and Epi-info (Centers for Disease Control and Prevention, Atlanta, GA) software.

## Results

In this study, 1005 patients met the inclusion and exclusion criteria in the study timeframe. The demographic characteristics of the study patients are listed in Table 3. 83.2% of the study patients were male, and 41% had identified comorbidities. 76.5% of patients underwent RSI. In 95.5% of the patients, no difficulty was encountered in airway management (Table 4). Commonly used pain management modalities after appendectomy in our patients were non-opioids (nonsteroidal anti-inflammatory drugs (NSAIDs), acetaminophen, local anesthetics (levobupivacaine), and regional anesthesia blocks (rectus abdominis plain block, transverse abdominis plain block, and combined) in 54.6%, and opioids in 45.4% (Table 4).

The total number of patients observed to have PPC in PACU was 27 (3.7%) out of 1005 (Table 5). While comparing the predisposing factors in terms of comorbidities (Table 6), a significant positive association was noted between the incidence of PPC and DM (16.4%) [OR 7.74 (95% CI: 1.61–37.2), p = 0.003]. Patients with BA also had a higher association with PPC [OR 3.46 (95% CI: 0.81–16.4), p = 0.07]. No significant association was found between other comorbidities and PPC. While analyzing the anesthesia procedure-related risk factors, the study found no significant association between the type of induction (RSI vs. non-RSI) and the incidence of PPC. There was a direct association between multiple intubation attempts (more than 2) and PPC, with an incidence of 9% [OR 3.76, 95% CI: 0.46–30.51, p = 0.004]. Laparoscopic surgeries were associated with a higher incidence of PPC than open procedures [OR 2.22, 95% CI: 0.88–5.6, P = 0.0001].

Intraoperative complications and events in PACU are described in Table 5, and multivariate logistic regression analysis of risk factors for PPC is shown in Table 6. The results showed that the use of total intravenous anesthesia (TIVA) is associated with a significantly higher odds ratio (OR) of 13.07 (with a confidence interval of 1.24–138.18) for PPC compared to inhalational plus intravenous anesthesia. Inhalational anesthesia alone had a lower OR of 1.07 (with a confidence interval of 0.47–2.43) than inhalational plus intravenous anesthesia. However, the differences between inhalational anesthesia and inhalational plus intravenous anesthesia were not statistically significant.

## Discussions

### Incidence of PPC

The incidence of PPC in our study was 3.7%. A comparable incidence is quoted in the existing literature, with a spectrum of results ranging from 0.04 to 23%.^4,9–13^ The cited data on the incidence of PPC encompasses various populations and surgical procedures, not specifically appendectomy patients. The wide range of reported PPC incidences in the literature suggests that factors such as patient population, surgery type, and individual characteristics contribute to the variability. Therefore, although the current study's PPC incidence falls within the reported range, it's crucial to recognize that the referenced data does not directly apply to the appendectomy population in this study.

### Gender distribution

In our study, the male-to-female ratio was 4.9:1. This higher male proportion is because of the population distribution of Qatar, where the predominant population in the age group of 15-50 years are expatriate male workers.^14,15^ Previous studies have shown a higher incidence of PPC in men than women,^10–12^, but we did not find a significant difference.

### Contributing factors to PPC

Airway instrumentation, multiple attempts, and assisted intubation techniques contribute to PPC. Difficult intubations were a predisposing factor for aspiration when performed electively by Sun et al.,^7^ thereby increasing the risk of PPC (67% risk in patients with no other prior risk factors). Proper assessment and adequate preparedness in airway management can considerably reduce multiple attempts and the risk of PCC.

### RSI v/s non-RSI techniques

Istvan et al.^8^ found considerable evidence in favor of RSI for reducing PPC. However, other authors have reported variable findings.^16,17^ We didn't find any significant association between the induction type and PPC incidence in our study (OR: 0.87). The equivalent risk of PPC in both techniques may be attributable to factors including appropriate patient selection, meticulous perioperative management, and standardized anesthesia protocols that ensure consistent patient management in both methods. Current clinical research debates various aspects of RSI, including cricoid pressure, intermittent positive pressure ventilation (IPPV), and medication dosage. Consequently, this finding may have contributed to the lack of significant clinical outcomes associated with RSI practice. In addition, the patient's NPO status during an emergency appendectomy may have contributed to the finding.

### Anesthesia maintenance method

The choice of anesthesia maintenance method can be crucial in reducing the risk of immediate PPC in this patient population. Healthcare providers should carefully consider the potential implications of utilizing TIVA versus inhalational plus intravenous anesthesia, particularly in high-risk patients or those prone to pulmonary complications. It is important to note that further research and larger-scale studies are warranted to validate these results and provide more robust evidence for guiding anesthesia practices. Nonetheless, these findings shed light on an essential aspect of perioperative care and emphasize the need for individualized anesthesia approaches to optimize patient outcomes.

### Association with DM

DM was associated with a 16.7% incidence of PPC compared to the general population, with an OR of 7.74 (95% CI: 1.61–37.2). In the study by Wang et al.,^10^ patients with DM posted for orthopedic and general surgery had a high incidence of PPC (36.6%) with a delay in extubation because of respiratory failure and respiratory muscle weakness. In a study on patients with DM undergoing coronary artery bypass surgery (CABG), Lauruschkat et al.^18^ observed similar outcomes. They found a significant need for reintubation and a need for ventilation for more than one day in the postoperative period in people with diabetes compared with nondiabetic patients (1.8% vs. 4.6%) and (4.8% vs. 9.9%), respectively. The extent of blood sugar control also determines the risk and degree of severity of pulmonary complications.18 Diabetes mellitus was shown to reduce FEV1 and FVC values as per the British Women's Heart and Health Study (1999–2001), leading to worsened clinical outcomes in the postoperative period.^19^ Though not assessed as an independent risk factor, deterioration of renal function and delayed gastric emptying increase the PPC,^6,19^ which is also seen in DM. Our study shows that even minor surgeries like an appendectomy predispose people with diabetes to a higher risk of PPC.

### Association with pre-existing pulmonary diseases

Pre-existing pulmonary diseases like smoking, COPD, URTI, and BA are associated with an increased risk of PPC.^4,6,11,12,20–24^ The pre-existing pulmonary co-morbidities predispose these patients to an increased tendency for bronchospasm, atelectasis, and pulmonary infections in response to airway instrumentation. In our study, BA was associated with an increased risk of PPC, with an OR of 3.65 (95% CI: 0.81–16.4), even though extensive data link smoking, pre-existing COPD, and URTI to PPC,^11,12,20–22^ no statistically significant risk was seen in our study. The absence of a statistically significant association does not necessarily imply the absence of an association. The sample size, selection bias, and other study limitations might influence the findings. The study population (15 to 50 years old and post-appendectomy) and methodological design (retrospective data review) may not have captured the full spectrum of PPC risk factors. Lastly, unmeasured confounding variables may have influenced the study's results. Overall, while it may be surprising that there was no statistically significant risk of PPC associated with smoking, pre-existing COPD, or URTI in the study, it is essential to remember that the results of a single study cannot be generalized and used to draw definitive conclusions. Additional research and meta-analyses are required to validate the findings and evaluate the study's robustness. Regional anesthesia techniques are usually beneficial to pulmonary function in response to pain. The chest wall and diaphragmatic dysfunction in response to pain can cause reduced pulmonary function.^25^

### Association with regional anesthesia and laparoscopic technique

Our study did not find a significant association between the type of regional anesthesia blocks for postoperative pain control and the incidence of PPC (Table 6). Our study found a positive association between PPC and laparoscopic technique [OR 2.2 (95% CI: 0.88–5.6)]. A previous study showed a lower risk of PPC in laparoscopic surgery than in open surgery.^26^ The difference could be because there are factors that favor and do not favor the development of PPC in laparoscopic surgeries. A smaller abdominal incision in laparoscopic surgery causes limited chest wall restriction, whereas there is an increased risk of pneumoperitoneum-associated diaphragmatic push-up and basal atelectasis. The favorable relationship between PPC and laparoscopic methods could be attributed to several variables. Creating a pneumoperitoneum, which is necessary for laparoscopic surgeries, is one of them. Numerous physiological changes brought on by the pneumoperitoneum, such as increased intra-abdominal pressure and reduced lung compliance, can make breathing harder for patients. Respiratory distress and a higher risk of PPC can result from this. There may be more causes for the link between PPC and laparoscopic surgery. For instance, laparoscopic surgery frequently requires extended anesthesia and operating time, which can raise the risk of PPC. Moreover, elderly patients or those with pre-existing respiratory issues may be more likely to undergo laparoscopic surgery, which can increase the PPC risk. Atelectasis was noted to occur more frequently in laparoscopic surgery by Jaschinski et al.^27^ We found a positive correlation between the surgery duration and PPC, supporting previous findings.^6,11,22,23^ A Fernandez-Bustamante et al.^13^ 2013 study established higher ASA physical status as a risk factor. Our study was restricted to ASA 1 and 2 status patients and was not designed to evaluate this effect. We did not find a significant difference between ASA 1 and 2.

## Limitations

1. Old data: Using samples from almost ten years ago is a significant limitation, as clinical practices and anesthesia management protocols may have changed over time, making it challenging to generalize the findings to current clinical practice.
2. Limited patient selection: The study included only ASA 1 or 2 patients aged 15-50 years, which may not represent the entire population of patients who undergo appendectomy under general anesthesia.
3. Retrospective study design: The accuracy and completeness of the medical records place restrictions on the study design, and potential inaccuracies or missing data may impact the findings.
4. Lack of standardized definitions: The study did not use standardized definitions for PPC, which may have resulted in inconsistencies in identifying and reporting outcomes.
5. Incomplete risk factor assessment: The study did not account for potential confounding factors such as body mass index, smoking history, or preoperative respiratory function tests. Therefore, whether the observed associations between risk factors and PPC are independent or confounded by other variables is still being determined. Age-related changes in respiratory physiology and diseases are confounding factors.
6. A control trial frame for the study would be better, but it would be difficult to conduct given this group's low frequency of PPC.

## Conclusions

The study shows that the incidence of PPC in young appendectomy patients with ASA 1 and 2 physical statuses is significant. The risk increases in those with BA, DM, and difficult airways requiring multiple intubation attempts. The RSI technique of anesthesia induction does not offer protection, whereas laparoscopic approaches and longer surgeries add to the risk of PPC.

## Patient Consent

A waiver of consent was obtained from the Institutional Review Board (IRB) as this was a retrospective analysis of patients' electronic medical records.

## Statement of Ethics

Ethical approval for this study (MRC/0895/2015) was provided by the Institutional Review Board (IRB) at the Medical Research Center (MRC) of Hamad Medical Corporation, Doha, Qatar (Chairperson: Dr. Ali Omrani), on 16th September 2015.

## Acknowledgments and Disclosures

Dr. Haitham Ibrahim Yakout Ibrahim, Dr. Mohamed Yagoub Abushora, Dr. Mohammad Azizuddin Imran, Dr. Rabia Basri, Dr. Michael Nabil Naseef Beshara, Ms. Mia Loren Gestopa Coyoca and Prof. Marcus Lance.

## Financial Support and Sponsorship

No financial support was received in the conduct of this study from any sources.

## Conflicts of Interest

The authors have no conflicts of interest to declare.

## Data Availability

Data supporting the study's conclusions is free of cost through open-access journals and websites.

## Author Contributions

**NK** – Mentor, Study concept and design, Data Analysis, Manuscript writing, and Manuscript editing. **MAMA** – Data collection, Data analysis, Literature review, Manuscript writing, and Manuscript editing. **CPKD** – Data collection, Data analysis, Literature review, Manuscript writing, and Manuscript editing. **PC** – Data analysis **MMA** – Data analysis, Literature review, Manuscript writing, and Manuscript editing. **TS** – Literature review, Manuscript writing, and Manuscript editing. **SS** – Manuscript writing and Manuscript editing.

## Figures and Tables

**Table 1.** Intraoperative parameters.

1. Type of induction	RSI vs. Non-RSI
2. Maintenance of anesthesia	Inhalation vs. TIVA
3. Intubation	Number of attempts
4. Difficult airway	Ease of Mask ventilation
	Use of adjuncts (stylet, bougie)
	Use of Video laryngoscopy
5. Intraoperative Complications	Aspiration
	Desaturation
	High intraoperative airway pressure
6. Neuromuscular monitoring use	Yes/no
7. Neuromuscular reversal agents	Neostigmine with atropine/glycopyrrolate/Sugammadex
8. Surgical techniques	Open vs. Laparoscopic
	Length of surgery
9. Use of opioids for postoperative pain	None
	Morphine
	Fentanyl
10. Use of regional anesthesia techniques:	None
	Local Wound Infiltration
	TAP block
	Rectus sheath block

RSI: Rapid sequence induction, TIVA: Total intravenous Anesthesia, TAP: Transversus Abdominis Plane.

**Table 2.** Postoperative parameters (PACU data).

1. Length of stay in PACU
2. Desaturation
3. Reintubation
4. Discharge with oxygen supplementation
5. Chest X-Ray performed

**Table 3.** Demographic Characteristics of study patients (Total number: 1,005 patients).

**Characteristics**	**N (%)**
**Gender**	
Male	836 (83.2%)
Female	169 (16.8%)
**Age**	
<30 years old	609 (60.6%)
>30 years old	396 (39.4%)
**ASA classification**	
ASA 1	575 (57.2%)
ASA 1	430 (42.8%)
**Fasting hours**	
<8 hours	35 (3.5%)
8-16 hours	788 (78.4%)
>16 hours	182 (18.1%)
**Presence of Comorbidities**	
No comorbidities	593 (59%)
With comorbidities	412 (41%)
**Type of comorbidities**	
URTI	4 (0.4%)
HTN	24 (2.4%)
DM	12 (1.2%)
Smoking	236 (23.5%)
Asthma	23 (2.3%)
Asthma + Smoking	3 (0.3%)
HTN + Smoking	6 (0.6%)
HTN + DM + Smoking	1 (0.1%)

N: number of patients, ASA: American Society of Anesthetists Physical Status classification, URTI: upper respiratory tract infection, HTN: Hypertension, DM: Diabetes Mellitus.

**Table 4.** Clinical and surgical characteristics of study patients (1,005 patients).

Characteristics	N (%)
**Type of induction**	
Classical	236 (23.5%)
RSI	769 (76.5%)
**Intubation attempts**	
1	965 (96%)
2	29 (2.9%)
>2	11 (1.1%)
**Maintenance of Anesthesia**	
Inhalational	639 (63.6%)
TIVA	4 (0.4%)
Inhalational + IV	362 (36%)
**Length of surgery**	
<1 hour	534 (53.1%)
1-2 hours	430 (42.8%)
>2 hours	41 (4.1%)
**Difficult airway**	
No difficulty	960 (95.5%)
Difficult mask ventilation	5 (0.5%)
Difficult intubation	16 (1.6%)
Devices used other than direct laryngoscopy	24 (2.4%)
**Devices used for intubation**	
Direct laryngoscopy alone	981 (97.6%)
Bougie	3 (0.3%)
Stylet	18 (1.8%)
Glideslope	3 (0.3%)
**NM monitoring devices**	
Used	47 (4.7%)
Not used	958 (95.3%)
**Reversal of NMB**	
Not used	172 (17.1%)
Neostigmine + glycopyrrolate	769 (76.5%)
Neostigmine + atropine	1 (0.1%)
Sugammadex	58 (5.8%)
Use of doxapram	2 (0.2%)
Neostigmine + glycopyrrolate, then Sugammadex	3 (0.3%)
**Postoperative opioids**	
Not used	549 (54.6%)
Morphine	408 (40.6%)
Fentanyl	6 (0.6%)
Others	42 (4.2%)
**Regional nerve block**	
Not used	388 (38.6%)
TAP block	206 (20.5%)
Rectus sheath block	65 (6.5%)
Local infiltration by the surgeon	342 (34%)
Others	4 (0.4%)
**Surgical technique**	
Open	408 (40.6%)
Laparoscopic	593 (59%)
Laparoscopic converted to open	4 (0.4%)

N: number of patients, RSI: rapid sequence induction, IV: intravenous, TIVA: total intravenous anesthesia, NM: neuromuscular, NMB: neuromuscular blockers, TAP: transversus abdominis plane.

**Table 5.** Intraoperative complications and Events in PACU (1,005 patients).

Events	N (%)
**Intraoperative complications**	
None	978 (97.3%)
Aspiration	1 (0.1%)
Desaturation	3 (0.3%)
High peak airway pressures	21 (2.1%)
Others*	2 (0.2%)
**Length of stay in PACU**	
<1 hour	349 (34.7%)
1-2 hours	623 (62%)
>2 hours	33 (3.3%)
**Events in PACU**	
None	978 (97.3%)
Reintubation	3 (0.3%)
Desaturation	2 (0.2%)
Discharged with oxygen	20 (2%)
Chest X-ray	2 (0.2%)

N: number of patients, PACU: post-anesthesia care unit, *: abnormal response to succinylcholine, effect ended after 80 minutes, the patient stayed intubated till full recovery of neuromuscular function.

**Table 6.** Multivariate logistic regression analysis of risk factors for PPC.

Variable	Postoperative Complication	No Postoperative Complication	P-value	Odds ratio (95% CI)
**Gender**				
Male	23 (2.8%)	813 (97.2%)	0.78	1.0 (Reference)*
Female	4 (2.4%)	165 (97.6%)		0.86 (0.29-2.51)
**Age**				
< 30 years old	14 (2.3%)	595 (97.7%)	0.35	1.0 (Reference)
>30 years old	13 (3.3%)	383 (96.7%)		1.44 (0.67-3.1)
**ASA**				
ASA 1	14 (2.4%)	561 (97.6%)	0.57	1.0 (Reference)
ASA 2	13 (3%)	417 (97%)		1.25 (0.58-2.69)
**Fasting hours**				
>16 hours	6 (3.3%)	176 (96.7%)	0.54	1.0 (Reference)
<8 hours	0 (0%)	35 (100%)		—
8-16 hours	21 (2.7%)	767 (97.3%)		0.8 (0.32-2.02)
**Presence of Comorbidities**				
No comorbidities	13 (2.2%)	580 (97.8%)	0.25	1.0 (Reference)
With comorbidities	14 (3.4%)	398 (96.6%)		1.57 (0.73-3.38)
**Type of Comorbidities**				
URTI	0 (0%)	4 (100%)	0.74	—
HTN	1 (4.2%)	23 (95.8%)	0.65	1.6 (0.21-12.28)
DM	2 (16.7%)	10 (83.3%)	0.003	7.74 (1.61-37.2)
Smoking	5 (2.1%)	231 (97.9%)	0.54	0.74 (0.28-1.96)
Asthma	2 (8.7%)	21 (91.3%)	0.07	3.65 (0.81-16.4)
Asthma + smoking	0 (0%)	3 (100%)	0.77	—
HTN + smoking	0 (0%)	6 (100%)	0.68	—
HTN + DM + smoking	0 (0%)	1 (100%)	0.87	—
**Type of induction**				
Classical	7 (3%)	229 (97%)	0.76	1.0 (Reference)
RSI	20 (2.6%)	749 (97.4%)		0.87 (0.37-2.09)
**Intubation attempts**				
1	25 (2.6%)	940 (7.4%)	0.004	1.0 (Reference)
2	1 (3.4%)	28 (96.6%)		1.34 (0.18-10.26)
>2	1 (9%)	10 (91%)		3.76 (0.46-30.51)
**Maintenance of Anesthesia**				
Inhalational + IV	9 (2.5%)	353 (97.5%)	0.02	1.0 (Reference)
TIVA	1 (25%)	3 (75%)		13.07 (1.24-138.18)
Inhalational	17 (2.7%)	622 (97.3%)		1.07 (0.47-2.43)
**Length of surgery**				
<1 hour	9 (1.7%)	525 (98.3%)	0.1	1.0 (Reference)
1-2 hours	16 (3.7%)	414 (96.3%)		2.25 (0.99-5.51)
>2 hours	2 (4.9%)	39 (95.1%)		2.99 (0.63-14.33)
**Difficult airway**				
No difficulty	26 (2.7%)	934 (97.3%)	0.85	1.0 (Reference)
Difficult mask ventilation	0 (0%)	5 (100%)		—
Difficult intubation	0 (0%)	16 (100%)		—
Devices used other than direct laryngoscopy	1 (4.2%)	23 (95.8%)		1.56 (0.2-12.01)
**Devices used for intubation**				
Direct laryngoscopy alone	26 (2.7%)	955 (97.3%)	0.01	1.0 (Reference)
Bougie	1 (33.3%)	2 (66.7%)		18.37 (1.61-209.0)
Stylet	0 (0%)	18 (100%)		—
glideslope	0 (0%)	3 (100%)		—
**NM monitoring devices**				
Used	2 (4.3%)	45 (95.7%)	0.5	1.0 (Reference)
Not used	25 (2.6%)	933 (97.4%)		0.60 (0.14-2.63)
**Reversal of NMB**				
Not used	1 (0.6%)	171 (99.4%)	0.3	1.0 (Reference)
Neostigmine + glycopyrrolate	26 (3.4%)	743 (96.6%)		5.98 (0.81-44.4)
Neostigmine + atropine	0 (0%)	1 (100%)		—
Sugammadex	0 (0%)	58 (100%)		—
Use of doxapram	0 (0%)	2 (100%)		—
Neostigmine + glycopyrrolate, then Sugammadex	0 (0%)	3 (100%)		—
**Post-operative opioids**				
Not used	10 (1.8%)	539 (98.2%)	0.25	1.0 (Reference)
Morphine	16 (3.9%)	392 (96.1%)		2.2 (0.99-4.9)
Fentanyl	0 (0%)	6 (100%)		—
Others	1 (2.4%)	41 (97.6%)		1.32 (0.16-10.52)
**Regional nerve block**				
Not used	15 (3.9%)	373 (96.1%)	0.17	1.0 (Reference)
TAP block	2 (1%)	204 (99%)		0.24 (0.55-1.08)
Rectus sheath block	0 (0%)	65 (100%)		—
Local infiltration by the surgeon	9 (2.9%)	332 (97.1%)		0.75 (0.33-1.69)
Others	0 (0%)	4 (100%)		—
**Surgical technique**				
Open	6 (1.5%)	402 (98.5%)	<0.0001	1.0 (Reference)
Laparoscopic	19 (3.2%)	574 (96.8%)		2.22 (0.88-5.6)
Laparoscopic converted to open	2 (50%)	2 (50%)		67.0 (8.05-557.77)
**Length of stay in PACU**				
<1 hour	4 (1.1%)	345 (98.9%)	0.04	1.0 (Reference)
1-2 hours	23 (3.7%)	600 (96.3%)		3.31 (1.13-9.64)
>2 hours	0 (0%)	33 (100%)		—

95% CI: 95% confidence interval, ASA: American Society of Anesthetist status classification, URTI: Upper respiratory tract infection, HTN: hypertension, DM: Diabetes Mellitus, RSI: rapid sequence induction, IV: intravenous, TIVA: Total Intravenous Anesthesia, NM: neuromuscular, NMB: neuromuscular blockers, TAP: transversus abdominis plane, PACU: post-anesthesia care unit, *(1.0 Reference): This group was used as a reference for calculating odds ratio, $: the absence of the specific type of comorbidities was used as a reference, -: no complications in that group, so odds ratio was not computed.
